# Electroretinographic assessment of rod- and cone-mediated bipolar cell pathways using flicker stimuli in mice

**DOI:** 10.1038/srep10731

**Published:** 2015-06-01

**Authors:** Naoyuki Tanimoto, Vithiyanjali Sothilingam, Mineo Kondo, Martin Biel, Peter Humphries, Mathias W. Seeliger

**Affiliations:** 1 Division of Ocular Neurodegeneration, Institute for Ophthalmic Research, Centre for Ophthalmology, Eberhard Karls University, Schleichstr. 4/3, D-72076 Tübingen, Germany; 2 Department of Ophthalmology, Mie University Graduate School of Medicine, 2-175 Edobashi, Tsu, Mie 514-8507, Japan; 3 Center for Integrated Protein Science Munich, CIPSM and Department of Pharmacy - Center for Drug Research, Ludwig-Maximilians-Universität München, Butenandtstr. 5-13, D-81377 München, Germany; 4 The Ocular Genetics Unit, Smurfit Institute of Genetics, Trinity College Dublin, Dublin 2, Ireland

## Abstract

Mouse full-field electroretinograms (ERGs) are dominated by responses of photoreceptors and depolarizing (ON-) bipolar cells, but not much of hyperpolarizing (OFF-) bipolar cells under conventional recording conditions. Here we investigate a novel ERG protocol in mice for functional assessment of the major ON- and OFF-bipolar cell pathways using flicker stimuli for a high luminance with varying frequency up to 30 Hz. Wild-type (WT) and functionally specific transgenic mice (*Cnga3*^-/-^, no cone photoreceptor function; *rho*^-/-^, no rod photoreceptor function; *mGluR6*^-/-^, no ON-bipolar cell function) were examined. The *Cnga3*^-/-^ flicker ERG was similar to the WT flicker ERG at very low stimulus frequencies, whereas ERGs were comparable between WT and *rho*^-/-^ mice at 5 Hz and above. Between 5 and 15 Hz, ERGs in *mGluR6*^-/-^ mice differed in configuration and amplitude from those in WT and *rho*^-/-^ mice; in contrast, response amplitudes above 15 Hz were comparable among WT, *rho*^-/-^ and *mGluR6*^-/-^ mice. In summary, we found three frequency ranges with these conditions that are dominated by activity in the rod pathways (below 5 Hz), cone ON-pathway (between 5 and 15 Hz), and cone OFF-pathway (above 15 Hz) that enables a quick overview of the functionality of the major bipolar cell pathways.

The full-field electroretinogram (ERG) is indispensable for retinal research. ERGs are typically elicited by a single or a repetitive light stimulation, so-called single-flash and flicker ERG, respectively. Single-flash ERGs are of great value, when they are recorded at a luminance series from (very) low to high under both dark-adapted and light-adapted conditions, allowing for an analysis of the functionality of certain neuronal components and systems, including the first, second and third order neurons of the retina^1^,^2^, as well as the rod and the cone photoreceptor pathways^3^. In experimental research using mouse models, flicker ERGs have been used rather for an examination to test a specific hypothesis than for a regular functional characterization; therefore, there is a variety of recording parameters and analytical methods, some of which may require additional equipment or software. On the one hand, tailoring protocols for a specific purpose is valuable and important; on the other hand, such protocols might be less compatible for more general purposes and could not easily be used in other laboratories.

This report examines a practical flicker ERG protocol for general functional phenotyping of mouse models that has three main features regarding availability and feasibility: First, the type of flicker stimulation, stimulus luminance, and frequency can be set by a commercially-available, ordinary ERG system, allowing the same protocol to be used in other laboratories without any additional expense and expertise. Second, the flicker protocol is short and can be used directly after a conventional dark-adapted single-flash luminance series before light-adapted experiments, i.e. it can be coupled with the regular single-flash experiments. Third, the response size is simply measured and analyzed without any mathematical treatment, allowing a quick diagnosis directly after or even during the recording. Another important aspect is the value of the recording protocol in terms of functional diagnostics; therefore, the question at the beginning of this study was whether this flicker protocol could provide any additional information about retinal function that is not assessable by single-flash ERGs only; for instance, OFF-cone bipolar cell (CBC)-associated responses because mouse full-field ERGs are dominated by responses of photoreceptors, rod bipolar cells (RBCs), and ON-CBCs, but not much of OFF-CBCs under conventional recording conditions.

Here we investigate the origin of flicker responses generated by the flicker ERG protocol in terms of rod- and cone-mediated bipolar cell pathways. To achieve this, we used pathway-specific knockout mouse models, allowing responses from specific pathway(s) to be isolated and directly visualized. In *Cnga3*^-/-^ mice, cones are dysfunctional due to a lack of the cone cyclic nucleotide-gated channels that stand at the end of the cone phototransduction cascade^4^; therefore, ERGs are generated exclusively by the rod pathways (i.e. five rod pathways: rod –RBC, rod – cone – ON-CBC, rod – cone – OFF-CBC, rod – ON-CBC, and rod – OFF-CBC)^5^,^6^,^7^. Conversely, in *rho*^-/-^ mice in which rod visual pigments are not produced^8^, rod-driven signals are completely abolished, and thus, only the cone pathways (i.e. two cone pathways: cone – ON-CBC, and cone – OFF-CBC) contribute to ERGs. Additionally, we used *mGluR6*^-/-^ mice lacking any light-evoked responses from ON-bipolar cells (i.e. RBC and ON-CBC)^9^, in order to isolate the rod and cone OFF-pathways (i.e. rod – cone – OFF-CBC, rod – OFF-CBC, and cone – OFF-CBC)^5^,^7^. Based on our ERG findings in these knockout mouse models, we could identify three frequency ranges according to the different pathway-contributions, including a cone OFF-pathway dominated frequency range that cannot be assessed in single-flash ERG experiments, and thus proving the value of the flicker ERG protocol for detailed functional diagnosis in mice.

## Results

### Dark-adapted single-flash ERG luminance series

As the major response components of the single-flash ERGs have been well investigated^1^, we could check the validity of ERG recordings on the basis of the single-flash ERG data, i.e. not only to confirm each functional phenotype, but also to rule out spontaneous abnormality which could affect ERGs, as well as to check general experimental conditions. The single-flash ERGs in each mouse line were consistent with those in the previous studies^4^,^8^,^9^,^10^.

**Figure 1:**
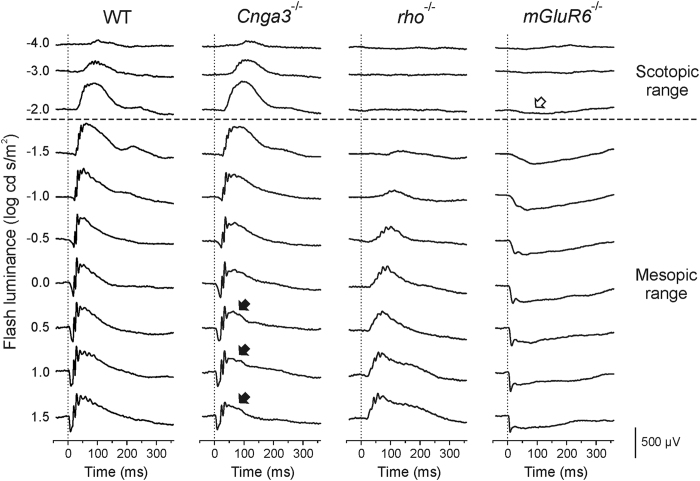
Representative dark-adapted single-flash electroretinograms (ERGs) of wild-type (WT), cone-specific CNGA3 channel knockout (*Cnga3*^-/-^), rod opsin knockout (*rho*^-/-^) and ON-bipolar cell-specific metabotropic glutamate receptor 6 knockout (*mGluR6*^-/-^) mice. Whereas ERGs up to -2.0 log cd s/m^2^ are derived fully by the rod pathways (scotopic range), those in the brighter luminance range are originated by both rod and cone photoreceptors (mesopic range). Due to the complete loss of signaling from cones in *Cnga3*^-/-^ mice, single-flash ERG signals are reduced on the top and on the trailing edge of the b-wave in the mesopic range (solid arrows). In *mGluR6*^-/-^ mice, the positive-going b-wave is completely missing; therefore, the negative-going response is fully visible, even at low stimulus luminances such as -2.0 log cd s/m^2^ (open arrow).

Several important features of the single-flash ERGs are described here briefly. Up to the luminance of -2.0 log cd s/m^2^, responses are comparable between wild-type (WT) and *Cnga3*^-/-^ mice, whereas there is no discernible response in *rho*^-/-^ mice, indicating that only rod photoreceptors are activated at those luminances, i.e. scotopic range (Fig. 1, above the horizontal dashed line). At higher luminances than -2.0 log cd s/m^2^, ERGs are detected in both *Cnga3*^-/-^ and *rho*^-/-^ mice, showing that both rod and cone photoreceptors are activated in the luminance range, i.e. mesopic range (Fig. 1, below the horizontal dashed line). In *Cnga3*^-/-^ mice, the top and the trailing edge of the b-wave are reduced in the mesopic range (Fig. 1, filled arrows), whereas the a-wave and the initial part of the b-wave are not affected, demonstrating a relatively slower timing of the cone pathway signalling under the given experimental conditions in mice. In *mGluR6*^-/-^ mice, the b-wave is completely defective because ON-bipolar cells do not generate any response after flash through knock-out of the ON-bipolar cell-specific mGluR6 glutamate receptor^9^. Therefore, a small, slow negative-going response is observable in *mGluR6*^-/-^ mice even at -2.0 log cd s/m^2^ (Fig. 1, open arrow), which is usually masked by the positive-going b-wave.

### Flicker ERG frequency series at the international standard flash luminance

**Figure 2:**
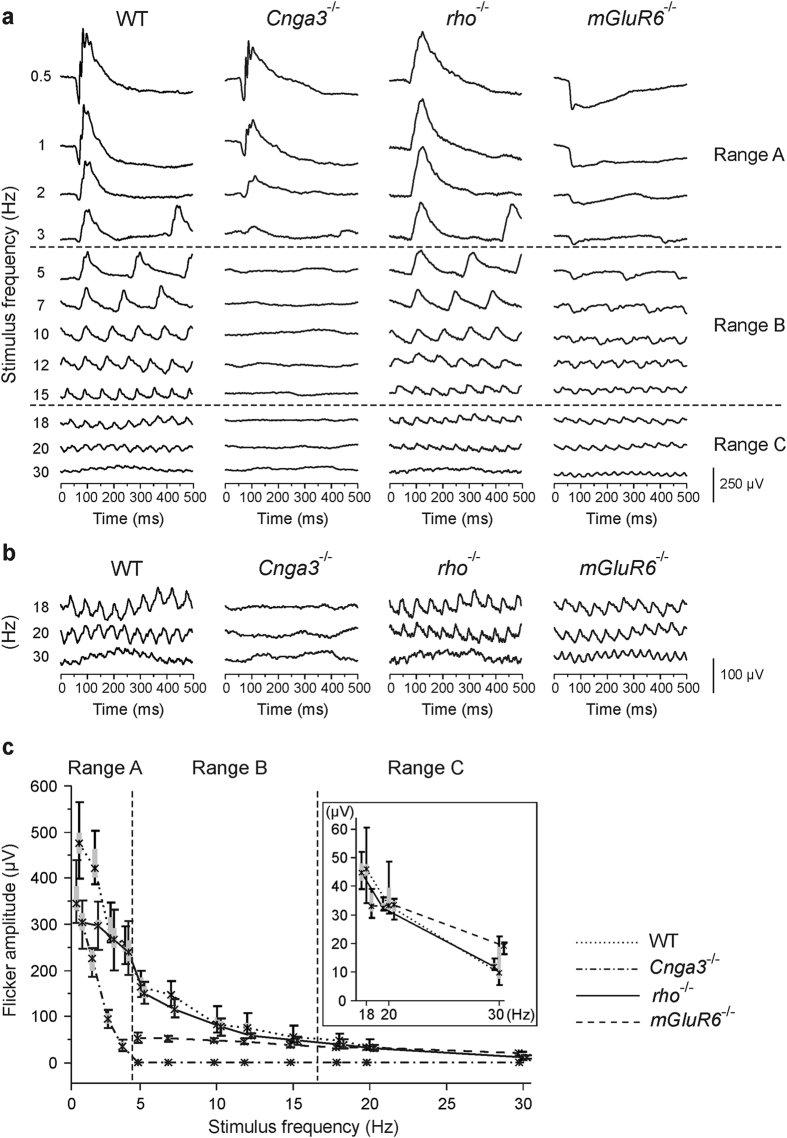
Flicker ERG frequency series at a fixed stimulus luminance of 0.5 log cd s/m^2^ without any background light. (**a**) Representative response traces in WT, *Cnga3*^-/-^, *rho*^-/-^, and *mGluR6*^-/-^ mice. (**b**) Enlargement of the response traces at 18, 20, and 30 Hz from (**a**). (**c**) Quantitative evaluation (box-and-whisker plot, indicating 5th, 25th, 50th, 75th, 95th percentiles of the data) of response amplitudes of WT, *Cnga3*^-/-^, *rho*^-/-^, and *mGluR6*^-/-^ mice. *Inset*: Details of the amplitude data of WT, *rho*^-/-^, and *mGluR6*^-/-^ mice at 18 Hz and above. The data were obtained from four mice (eight eyes) in *Cnga3*^-/-^, *rho*^-/-^ and *mGluR6*^-/-^, and from three WT mice (six eyes). In *mGluR6*^-/-^ mice, response amplitudes were measured at 5 Hz and above, but not below 5 Hz, because there was no clear positive-going response below 5 Hz. On the basis of response amplitude and shape in three pathway-specific knockout mouse models, the flicker ERG series could be divided into three distinct frequency ranges that are dominated by activity in the rod pathways (range A; below 5 Hz), cone ON-pathway (range B; 5–15 Hz), and cone OFF-pathway activity (range C; above 15 Hz).

Figure 2 shows a representative series of the flicker ERGs in each mouse line and a summary of amplitude data. In WT mice, the response at 0.5 Hz was very similar to the dark-adapted single-flash ERG at the same luminance in terms of the existence of three response components which correspond to the a-wave, the b-wave, and the oscillatory potentials (OPs) of the single-flash ERG (Fig. 2a, top of the left column). Similarly, in each mutant mouse model, the response property at 0.5 Hz was comparable to that of the corresponding single-flash ERG: predominant signal reduction of the top and the trailing edge of the positive-going response in *Cnga3*^-/-^ mice, no substantial negative-going response in *rho*^-/-^ mice, and complete lack of the positive-going response and the oscillations in *mGluR6*^-/-^ mice (Fig. 2a, top).

The shape of the WT flicker response changed with increasing flicker frequency: response components analogous to the a-wave and the OPs became smaller and merged together with the b-wave analogue even at low flicker frequencies, and at middle and high stimulus frequencies each flicker response had a relatively simple triangle shape (Fig. 2a, left column). Accompanied by the change of the shape, the size of the response became smaller, and at 30 Hz, there remained only small steady-state flicker responses in WT mice (Fig. 2a, bottom of the left column; Fig. 2b, left column; Fig. 2c).

At low stimulus frequencies, such as 0.5 and 1 Hz, flicker ERGs were similar in shape between WT and *Cnga3*^-/-^ mice, but not between WT and *rho*^-/-^ mice (Fig. 2a, top), indicating that WT flicker responses at those frequencies are dominated by signals from the rod pathways. In *Cnga3*^-/-^ mice, the flicker amplitude decreased with increasing stimulus frequency much faster than the WT flicker amplitude; as a result, the flicker response vanished completely at 5 Hz under the given conditions. Below 5 Hz, flicker ERGs were observed in both *Cnga3*^-/-^ and *rho*^-/-^ mice, demonstrating that flicker responses below 5 Hz are generated by both rod and cone pathways (mesopic). At 5 Hz and above, no steady-state flicker response was evoked in *Cnga3*^-/-^ mice, whereas response amplitudes were comparable between WT and *rho*^-/-^ mice (Fig. 2c), indicating that any steady-state response at 5 Hz and above is purely cone-driven and does not contain any signals originated from rod photoreceptors. We thus determined the rod pathway-dominant, mesopic frequency range below 5 Hz (Fig. 2a,c, range A) and the cone pathway-specific frequencies (5 Hz and above) at the luminance of 0.5 log cd s/m^2^.

Next, we compared the cone pathway-specific responses at 5 Hz and above in WT and *rho*^-/-^ mice with those in *mGluR6*^-/-^ mice in order to explore contributions of the cone ON- and the cone OFF-pathway to the flicker ERGs. At 5, 7, and 10 Hz, flicker responses in *mGluR6*^-/-^ mice did not show any clear positive-going response peak which was evident in WT and *rho*^-/-^ mice (Fig. 2a). Therefore, the responses in *mGluR6*^-/-^ mice were considerably smaller than those in WT and *rho*^-/-^ mice (Fig. 2c), demonstrating that they were dominated by cone ON-pathway activities. The difference in response amplitude became smaller at 12 and 15 Hz; however, the response shape in *mGluR6*^-/-^ mice was still trapezoid due to a lack of positive-going response peak (Fig. 2a). This differed from the triangle shape of WT and *rho*^-/-^ responses, indicating that the cone ON-pathway still had a predominant influence on the responses at 12 and 15 Hz. Notably, both the shape and the size of each flicker response became comparable at 18 Hz and above among WT, *rho*^-/-^, and *mGluR6*^-/-^ mice (Fig. 2a,b; Fig. 2c, inset), indicating that the flicker responses were highly dominated by cone OFF-pathway activities. Therefore, we determined the frequency border dividing the cone pathway-specific frequencies into two ranges: range B (between 5 and 15 Hz) and range C (above 15 Hz), dominated by activities in the cone ON- and OFF-pathway, respectively (Fig. 2a,c). Taken together, our results clearly demonstrated that, in the flicker ERG frequency series using 0.5 log cd s/m^2^ stimuli, the functional status of the major photoreceptor – bipolar cell pathways can be overviewed in a single recording session.

## Discussion

In this study, we evaluated a flicker ERG frequency series at the luminance of 0.5 log cd s/m^2^ in mice by measuring pathway-specific mouse models and found that the stimulus frequencies (0.5–30 Hz) could be divided into three distinct ranges on the basis of photoreceptor – bipolar cell pathway contributions to flicker ERGs (Fig. 2a,c). The border between the rod pathway-dominant mesopic frequencies (below 5 Hz) and the cone pathway-specific frequencies (at 5 Hz and above) confirmed the results of our previous study^3^. It is noted that the flicker fusion frequency of the rod-driven signals depends on the flash luminance, e.g. around 18 Hz at -2.0 log cd s/m^2^ luminance^11^, which differs 5 Hz at 0.5 log cd s/m^2^ in this study. We additionally demonstrated that the flicker ERGs in *mGluR6*^-/-^ mice lacking any response from ON-bipolar cells were comparable to those in *rho*^-/-^ and WT mice at high stimulus frequencies above 15 Hz, indicating that the cone ON-pathway does not contribute substantially to the response amplitudes and that the cone OFF-pathway is necessary for generation of high frequency steady-state flicker responses (Fig. 2a,c, range C). Actually, OFF-CBCs receive not only cone photoreceptor signals but also rod photoreceptor signals via cone pedicles through gap junctions between rod and cone photoreceptors, or directly from rod photoreceptors^5^,^7^. However, contributions of rod-driven signals to the steady-state flicker ERGs in range C are unlikely under the given experimental conditions because the functionally rod-specific *Cnga3*^-/-^ mice do not generate any response in range C (Fig. 2); thus, the flicker responses in range C should be purely cone-driven, reflecting functionality of the cone OFF-pathway (cone – OFF-CBC). It is noted that flicker responses are generated by interactions between size and phase of responses of cones, ON-CBCs, and OFF-CBCs under light-adapted conditions that was first demonstrated in monkeys^12^,^13^ and later analysed also in mice^14^,^15^. Receptoral component is relatively large at very low flicker frequencies and decreases with increasing frequency; as a result, flicker ERGs at high flicker frequencies are dominated by post-receptoral components^15^. In addition, amplitude and phase differences are quite small at around 15-16 Hz and above between control and *nob* mice (mice with absent RBC and ON-CBC activity^16^), indicating predominant OFF-CBC contributions to high frequency flicker ERGs^14^,^15^. Therefore, the finding of range C in this study were in line with those studies.

The flicker ERG frequency series has several positive features regarding availability and feasibility: 1) The same stimulus and recording parameters can be set using a commercially-available, ordinary ERG system complying with the International Society for Clinical Electrophysiology of Vision standard for full-field clinical ERG^17^,^18^. Therefore, the flicker ERG data in other laboratories may be interpreted as described in this manuscript without testing functionally-specific mouse models in each laboratory again. 2) The flicker recording takes less than four minutes and can be incorporated between the conventional dark-adapted and light-adapted single-flash ERG luminance series, allowing for a reliable, systematic assessment of single-flash and flicker data obtained successively using a single anaesthesia. This recording paradigm could be applied well for longitudinal time-course studies as mice have to be anaesthetized only once for each time point. 3) An overview of the functional status of the rod- and cone-mediated bipolar cell pathways can be obtained quickly without any background light and mathematical treatment, which may facilitate functional diagnostics as well as smooth planning of other experiments, e.g. generating “tailor-made” ERG protocols for an in-depth functional characterization, and planning/performing *ex vivo* experiments in the same animals. 4) The *in vivo* functional evaluation of the cone OFF-pathway presented here is especially valuable because traditional methods cannot be used in mice: The cone OFF-pathway is typically evaluated with long-flash ERG under light-adapted conditions in humans and non-human primates; however, in mice the OFF response at stimulus offset is not evident in long-flash ERG recordings^1^,^15^,^19^.

There are at least three conditions under which responses of the flicker ERG frequency series can not be interpreted according to the frequency ranges A, B, and C determined in this study. First, ERG responses generally depend on stimulus luminance^1^; therefore, flicker responses obtained from albino mice using the same recording parameters may have other properties. Second, the frequency border between ranges A and B (with and without rod pathway contributions, respectively), may be shifted when photoreceptors are desensitized due to a low amount of 11-cis-retinal, e.g. in cases with a dysfunctional visual cycle^20^,^21^. Third, cone pathway-specific flicker responses in both ranges B and C may be extraordinarily suppressed when signals generated by rod photoreceptors are remarkably prolonged - which may affect cone pathway signalling owing to the convergence of rod and cone pathway signals^22^.

In summary, the flicker protocol provides a quick overview of the functionality of the major photoreceptor – bipolar cell pathways of the mouse retina, including the cone OFF-pathway. As conventional single-flash ERGs are highly dominated by responses of ON-bipolar cell pathways and contain information about layers (outer and inner retina), flicker ERGs of our recording protocol could complement single-flash ERGs in mice, enabling an in-depth functional characterization of mouse models and a discrimination of underlying functional pathologies. Due to the short recording time, the flicker protocol could also be used clinically in humans. However, the human B and C ranges remain to be determined owing to the different timing in cone pathway signalling.

## Methods

### Ethical approval

All animals were treated in accordance with the ARVO statement for the Use of Animals in Ophthalmic and Vision Research and the law of animal experimentation issued by the German Government. All experimental procedures were approved by the local government authority (Regierungspräsidium Tübingen).

### Animals

For this work, mice from the following lines were used in the experiments: mice lacking cone photoreceptor function (*Cnga3*^-/-^, cone CNG channel deficient)^4^, mice devoid of rod photoreceptor function (*rho*^-/-^, rod opsin knockout)^8^, and mice lacking ON-bipolar cell function (*mGluR6*^-/-^, ON-bipolar cell-specific metabotropic glutamate receptor 6 knockout)^9^, and WT (C57BL/6J) mice. All mice were examined at the age of 4–5 weeks, which is the ideal time window in order to analyze a primary functional change due to a genetic defect in mice, because retinal development is usually complete up to the age but no secondary change, e.g. degeneration, has yet taken place in most cases, even when rod outer segments are completely abolished, e.g. in *rho*^-/-^ mice^10^. Note that there are a few exceptional cases, such as the *rd1* mouse, in which retinal degeneration starts very early and the outer retina is destroyed already at 4–5 weeks of age^23^.

### Electroretinography

ERGs were recorded as described in the following sections; for additional details see our previous publications^3^,^24^. Briefly, ERG experiments were performed with a full-field Xenon flash system, which consisted of a light source for stimulation, a Ganzfeld bowl, a signal amplification system, a PC-based control and recording unit, and a monitor screen (Multiliner Vision, VIASYS Healthcare GmbH, Höchberg, Germany).

Mice were dark-adapted overnight before the experiments and anesthetized with subcutaneous injection of a mixture of ketamine, xylazine, and physiological saline. Ketamine and xylazine were given 66.7 mg/kg body weight and 11.7 mg/kg body weight, respectively. The pupils were dilated with tropicamide eye drops (0.5%; Mydriaticum Stulln, Pharma Stulln, Stulln, Germany). Gold wire ring electrodes (active electrodes) were moistened with methylcellulose and positioned on the surface of both corneae for binocular ERG recordings. Stainless steel needle electrodes were applied subcutaneously at the middle of the forehead region and the back near the tail as a reference and a ground electrode, respectively.

### Single-flash ERG experiments

Single-flash ERGs were obtained under dark-adapted conditions (no background illumination, 0 cd/m^2^). Single white-flash stimuli ranged from -4 to 1.5 log cd s/m^2^, divided into ten steps. Ten responses were averaged with interstimulus intervals of 5 s (for -4 to -0.5 log cd s/m^2^) or 17 s (for 0 to 1.5 log cd s/m^2^). Band-pass filter frequencies were 0.3 and 300 Hz.

### Flicker ERG experiments

Responses to trains of brief flashes for a fixed luminance (0.5 log cd s/m^2^; the International Society for Clinical Electrophysiology of Vision standard flash luminance)^17^ with varying frequency (0.5, 1, 2, 3, 5, 7, 10, 12, 15, 18, 20 and 30 Hz) were obtained without any background illumination (0 cd/m^2^). The luminance was chosen because it was bright enough to stimulate both rod and cone photoreceptors, i.e. high mesopic stimulus luminance, as well as because most commercially-available ERG systems are feasible to generate light stimuli at the luminance up to the frequency of 30 Hz due to the long-existing international standard established firstly in 1989^25^. We did not use any background illumination in order to assess a transition from rod pathway-dominated to cone pathway-specific activities^3^, allowing dark- and light-adapted single-flash ERG data to be compared and assessed with flicker ERG data for functional diagnosis. Flicker responses were averaged either 20 times (for 0.5, 1, 2, and 3 Hz) or 30 times (for 5 Hz and above) over time, i.e. steady-state flicker ERGs. Band-pass filter frequencies were 0.3 and 300 Hz as those for the single-flash experiments. All recording parameters were pre-programmed; thus, the inter-recording interval was less than five seconds, allowing the whole recording session to be performed within four minutes. Flicker response amplitudes were measured from the trough to the peak of each response at all frequencies in WT, *Cnga3*^-/-^, and *rho*^-/-^ mice. In *mGluR6*^-/-^ mice, response amplitudes were measured at 5 Hz and above, but not below 5 Hz, because there was no clear positive-going response below 5 Hz.

The flicker ERG frequency series was started approximately 30 seconds after the end of the preceding single-flash ERG luminance series in all mice because the stimulus luminance of the flicker ERG protocol was 1 log unit lower than that of the last single-flash ERG recording. This inter-protocol interval was enough for reproducible steady-state flicker ERGs, which was important especially for low stimulus frequencies.

## Additional Information

**How to cite this article**: Tanimoto, N. *et al.* Electroretinographic assessment of rod- and cone-mediated bipolar cell pathways using flicker stimuli in mice. *Sci. Rep.* **5**, 10731; doi: 10.1038/srep10731 (2015).
